# Ergosterone-coupled Triazol molecules trigger mitochondrial dysfunction, oxidative stress, and acidocalcisomal Ca^2+^ release in *Leishmania mexicana* promastigotes

**DOI:** 10.15698/mic2016.01.471

**Published:** 2015-12-11

**Authors:** K Figarella, S Marsiccobetre, I Arocha, W Colina, M Hasegawa, M Rodriguez, A Rodriguez-Acosta, M Duszenko, G Benaim, NL Uzcategui

**Affiliations:** 1 Laboratory of Genomics and Proteomics, Biotechnology Center, IDEA Foundation. Caracas, Venezuela.; 2 Laboratory of Natural Products, School of Chemistry, Central University of Venezuela, Venezuela.; 3 Laboratory of Immunochemistry and Ultrastructure, Institute for Anatomy, Central University of Venezuela, Venezuela.; 4 Laboratory of Molecular Parasitology, Interfaculty Institute for Biochemistry, Tuebingen University, Germany.; 5 Laboratorio de Señalización Celular y Bioquímica de Parásitos, Instituto de Estudios Avanzados (IDEA) and Instituto de Biología Experimental, Facultad de Ciencias. Universidad Central de Venezuela, Caracas, Venezuela.

**Keywords:** Leishmania, ergosterol, azoles, cell death, autophagy, ROS, Ca^2+^

## Abstract

The protozoan parasite *Leishmania* causes a variety of sicknesses with different clinical manifestations known as leishmaniasis. The chemotherapy currently in use is not adequate because of their side effects, resistance occurrence, and recurrences. Investigations looking for new targets or new active molecules focus mainly on the disruption of parasite specific pathways. In this sense, ergosterol biosynthesis is one of the most attractive because it does not occur in mammals. Here, we report the synthesis of ergosterone coupled molecules and the characterization of their biological activity on *Leishmania mexicana* promastigotes. Molecule synthesis involved three steps: ergosterone formation using Jones oxidation, synthesis of Girard reagents, and coupling reaction. All compounds were obtained in good yield and high purity. Results show that ergosterone-triazol molecules (Erg-GTr and Erg-GTr_2_) exhibit an antiproliferative effect in low micromolar range with a selectivity index ~10 when compared to human dermic fibroblasts. Addition of Erg-GTr or Erg-GTr_2 _to parasites led to a rapid [Ca^2+^]_cyt_ increase and acidocalcisomes alkalinization, indicating that Ca^2+^ was released from this organelle. Evaluation of cell death markers revealed some apoptosis-like indicators, as phosphatidylserine exposure, DNA damage, and cytosolic vacuolization and autophagy exacerbation. Furthermore, mitochondrion hyperpolarization and superoxide production increase were detected already 6 hours after drug addition, denoting that oxidative stress is implicated in triggering the observed phenotype. Taken together our results indicate that ergosterone-triazol coupled molecules induce a regulated cell death process in the parasite and may represent starting point molecules in the search of new chemotherapeutic agents to combat leishmaniasis.

## INTRODUCTION

Leishmaniasis is a group of diseases caused by different species of parasites belonging to the *Trypanosomatidae* family. Depending on the strain involved, it may lead to self-healing cutaneous lesions, mucocutaneous lesions or severe/fatal generalized visceral infection. The World Health Organization considers it as one of the most important tropical diseases in the world. Currently the overall prevalence of human infection is estimated to be at 12 million cases and approximately 350 million people are at risk of contracting infection 1,2. Specific treatments for this disease remain unsatisfactory due to the limited efficacy of current drugs, and frequent deleterious side effects. Current treatments are very costly, long-lasting and have harmful effects. They are based on pentavalent antimonials (Glucantime and Pentostam), although its use is empiric and the mechanism of action is not well understood. Occurrence of resistance against the two have led to other formulations, such as Amphotericin or Miltefosine, which also present some limitations like failure to treat some cases of visceral leishmaniasis or its teratogenicity and pharmacokinetics (slow elimination from the body, which may also induce resistance), respectively 3. Under these circumstances, investigations that contribute to the development of new, more efficient and selective drugs against the parasite are needed.

The search for new molecules has been directed to parasite specific targets or pathways. In this sense, the ergosterol biosynthesis, which is restricted to fungi and trypanosomatids, has been an attractive focus of attention for chemotherapy. Ergosterol is an essential component of parasite membranes 4. Its synthesis can be inhibited by azoles, which act on the 14-α-metyl-lanosterol-demethylase enzyme diminishing the formation of the ergosterol precursor lanosterol 5. Many studies have been focused on designing inhibitors of ergosterol biosynthesis. In an attempt to take advantage of the antileishmanial potency of triazol for the development of drug candidates with improved activity against leishmaniasis, we synthetized ergosterone-triazol hybrid molecules and evaluated their effect on the survival and intracellular Ca^2+^ mobilization of *Leishmania (L) mexicana* promastigotes. To obtain an understanding of the antileishmanial mechanism of action of ergosterone-triazol coupled molecules, cell death parameters were assayed. The findings offer further insight into the effects of triazol on Leishmania and point toward a new strategy for the development of antileishmanial drugs.

## RESULTS

### Compund synthesis

**Figure 1:**
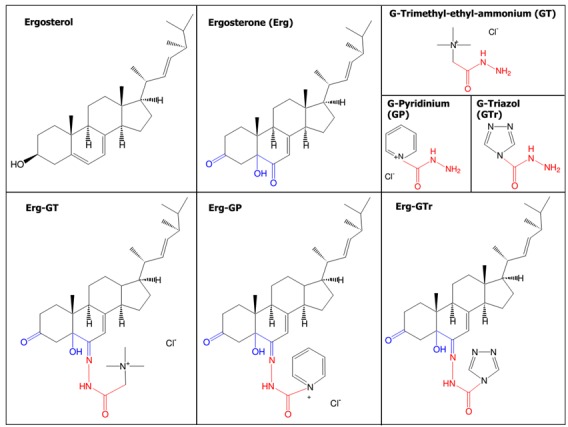
FIGURE 1: Structures of the compounds evaluated on *Leishmania mexicana* promastigotes. IUPAC names: **Ergosterol**: 10,13-dimethyl-17-(1,4,5-trimethyl-hex-2-enyl)-2,3,4,9,10,11,12,13,14,15,16,17-dodecahydro-1H-cyclopenta[a]phenanthren-3-ol; **Ergosterone (Erg)**: 5-hydroxy-10,13-dimethyl-17-(1,4,5-trimethyl-hex-2-enyl)-1,4,5,9,10,11,12,13,14,15,16,17-dodecahydro-2H-cyclopenta[a]phenanthrene-3,6-dione; **GT**: hydrazinocarbonylmethyl-trimethyl-ammonium chloride; **GP**: 1-hydrazinocarbonyl-pyridinium chloride; **GTr**: [1,2,4]triazole-4-carboxylic acid hydrazide; **Erg-GT**: [5-hydroxy-10-methyl-3-oxo-17-(1,4,5-trimethyl-hex-2-enyl)-1,2,3,4,5,9,10,11,12,13,14,15,16,17-tetradecahydro-cyclopenta[a]phenanthren-6-ylidene-hydrazinocarbonylmethyl]-trimethyl-ammonium chloride; **Erg-GP**: 1-[5-hydroxy-10-methyl-3-oxo-17-(1,4,5-trimethyl-hex-2-enyl)-1,2,3,4,5,9,10,11,12,13,14,15,16,17-tetradecahydro-cyclopenta[a]phenanthren-6-ylidene-hydrazinocarbonyl]-pyridinium chloride; **Erg-GTr**: [1,2,4]triazole-4-carboxylic acid [5-hydroxy-10,13-dimethyl-3-oxo-17-(1,4,5-trimethyl-hex-2-enyl)-1,2,3,4,5,9,10,11,12,13,14,15,16,17-tetradecahydro-cyclopenta[a]phenanthren-6-ylidene]-hydrazide. The global yield of the synthesis was 49%, 45%, and 49% for Erg-GTr, Erg-GT, and Erg-GP, respectively.

Compunds evaluated in this work were synthetized starting from commercially available ergosterol (5,7,22-Ergostatrien-3beta-ol). Ergosterol was oxidized using the Jones reactive, consisting of chromic anhydride and sulfuric acid 6. The reaction product was purified by column chromatography on alumina using a mixture 9.7:0.3 dichloromethane/methanol. Following evaporation of the solvent a light yellow solid was obtained with a yield of 54% (mp 266-268°C). Using spectroscopic techniques (NMR ^1^H and ^13^C), the reaction product obtained was elucidated as 7,22-ergostadien-5α-ol-3,6-dione (ergosterone) (Erg). To couple triazol to ergosterone, the Girard reagent principle was used 7. First, ethyl chloroacetate reacted with nitrogen containing compounds (triazol in our case) to obtain the respective quaternary salts and second, these salts reacted with hydrated hydrazine to produce the corresponding Girard reagent (Girard-triazol) (GTr). The obtained solid was recrystallized in methanol. The yield of this reaction was 67% (mp 84-86°C). Ergosterone coupled molecules were synthetized in a reaction with equimolar substrate amounts and using methanol, under reflux conditions and acetone as desiccant. Under these conditions Erg was linked to GTr in a very efficient reaction with a yield of 91%. In order to study the biological activity of different ergosterone derivatives, two other commercially available Girard reagents, Girard-pyridine (GP) and Girard-trimethylethylammonium (GT), were also coupled to ergosterone and analyzed. Again, reaction products were obtained in high yield, i.e. 91% and 84% for Erg-GP and Erg-GT, respectively. Derivatives were also obtained as di-substituted compounds (C3 and C6 Girard T, P or Tr derivatives), by doubling the molar ratio of Girard reagents. The NMR analysis of all synthesized compounds showed that they were efficiently obtained with a purity of > 97%. All molecules described are shown in Figure 1. They were tested on *L. mexicana* promastigotes and human dermic fibroblasts. Results of their half-inhibitory concentration (IC_50_) are resumed in Table 1. All compounds were soluble in DMSO and stable for at least 6 months in solution.

### Biological effects of ergosterone coupled molecules on *Leishmania mexicana* promastigotes and human fibroblasts

Parasites were treated with different concentrations of the synthesized compounds for 48 hours to determine their cytotoxic effect. A dose dependent growth inhibition was observed after this time. Oxidation of ergosterol to ergosterone increased growth inhibition capability on promastigotes, while Girard compounds lacking ergosterone (GP, GT, and GTr) did not show inhibition at concentrations even higher than 100 μM. Ergosterone coupled molecules exhibited higher cytotoxic effects than its component molecules alone itself (Table 1). Interestingly, mono- or di-substituted coupled compounds containing triazol (Tr) were not only more cytotoxic but also more selective against the parasite. In fact, Erg-GTr and Erg-GTr_2_, displayed the smallest half-inhibitory concentration (IC_50_) values and were almost 10 times more effective on promastigotes than on human dermic fibroblasts.

**Table 1:** Half-inhibitory concentration (IC_50_) of ergosterone coupled molecules on *Leishmania mexicana* promastigotes and human dermic fibroblasts. Concentrations are given in μM. nd: not determined.

**Compound**	**IC_50_ on *L. mexicana* promastigotes**	**Selectivity index**	**IC_50_ on fibroblasts**
Ergosterol	33.8 ± 4.7	0.8	27.0 ± 3.5
Ergosterone	9.0 ± 0.7	3.1	28.3 ± 8.5
GT	>200	-	nd
GP	>200	-	nd
GTr	158.3	-	nd
Erg-GT	10.4 ± 0.2	3.5	36.4
Erg-GT_2_	6.5 ± 2.5	5.5	35.6
Erg-GP	42.4 ± 2.8	1.0	44.5
Erg-GP_2_	6.2 ± 1.3	3.8	23.7
Erg-GTr	1.4 ± 0.4	9.8	13.7 ± 1.9
Erg-GTr_2_	2.3 ± 0.5	7.7	17.6 ± 0.3

### Phosphatidylserine exposure after Erg-GTr and Erg-GTr_2_ treatment

**Figure 2:**
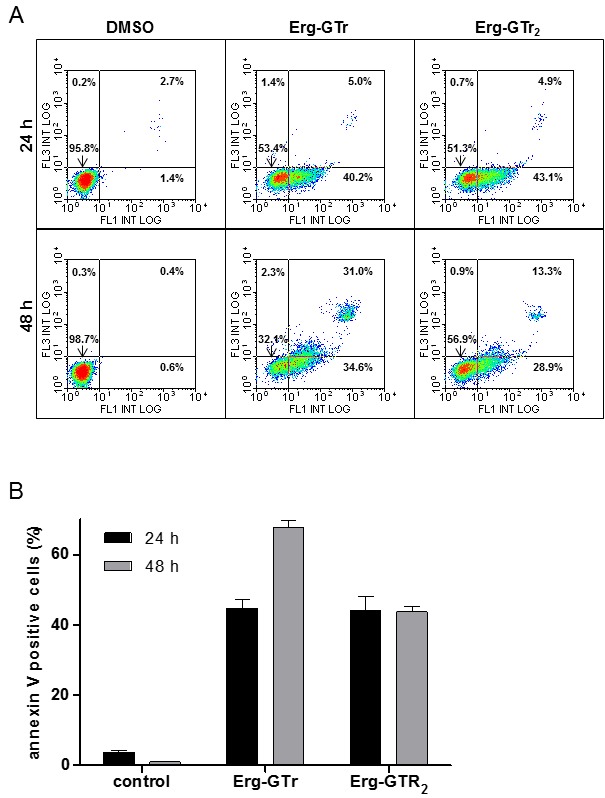
FIGURE 2: Phosphatidylserine exposure after Erg-GTr treatment. **(A) **Double staining propidium iodide (PI) and Annexin V-Alexa 488 from parasites after 24 and 48 hours treatment; left column control cells, middle and right column experimental cells. The dot plots represent the results of a typical experiment. **(B)** Total population in percentage exposing phosphatidylserine after the indicated time. Bars show the mean +/- SD of at least three independent experiments. The percentages were determined using the WinMDI software.

The effect of Erg-GTr and Erg-GTr_2_ was further analyzed to determine the cell death mechanism. Asymmetry of the plasma membrane is loosed when cell death processes are activated. The Annexin V-AlexaFluor488/propidium iodide double staining was used to detect phosphatidylserine (PS) in the outer leaflet of the plasma membrane using flow cytometry after parasite treatment. Cells were incubated for 24 and 48 hours with Erg-GTr (1.4 μM) or Erg-GTr_2_ (2.3 μM). As shown in figure 2A, after 24 hours incubation, Erg-Tr and Erg-GTr_2_ caused a PS exposure in 40.2% or 43.1% of the cell population, respectively. At this time, the integrity of the plasma membrane was still intact, as evidenced by a low PI staining (5% or lower). After 48 hours, the percentage of Annexin V positive cells increased to reach more than 60% (Erg-GTr) or was maintained at around 40% in the case of Erg-GTr_2_ (Figure 2B). The number of PI positive cells increased to 31% and 13.3% after 48 hours incubation with Erg-GTr and Erg-GTr_2_, respectively. This phenomenon has been reported as a criterium of the late stage of cell death processes 8.

### Parasite mitochondrion becomes hyperpolarized after ergosterone-triazol treatment

**Figure 3:**
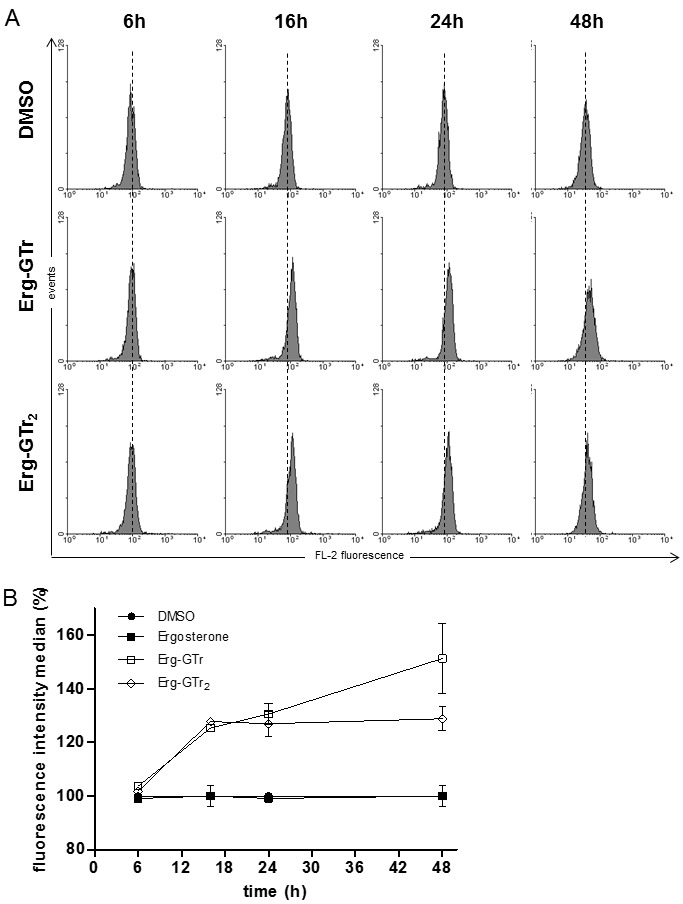
FIGURE 3: Mitochondrial membrane potential after Erg-GTr treatment. **(A)** Histograms showing fluorescence intensity of parasites stained with Mitotraker Red CMXROS. Dashed line represents the histogram median of control cells. Right displacement indicates hyperpolarization. Histograms represent one of at least three experiments that showed the same tendency. **(B)** Quantification of the fluorescence intensity median versus time. Changes in the fluorescence were calculated and represented in percentage, where the median values of control parasites means 100% fluorescence. Results show the mean +/- SD of three independent experiments.

In order to determine the energetic state of the mitochondrion after parasites treatment, the compound Mitotraker Red CMXROS was used. As described in the Materials and Methods section, parasites were treated for 6, 16, 24, and 48 hours with each molecule at its IC_50_, then incubated with Mitotraker Red CMXROS (50 nM) for 30 minutes under culture conditions, and finally analyzed by flow cytometry. Figure 3A shows representative histograms obtained for control and treated cells. Histograms of treated parasites displayed higher fluorescence intensities than those of control cells as evidenced by its right displacement. Analysis of the histogram fluorescence intensity revealed that after 16 hours treatment the histogram median in treated cells increased to 25%, reaching up to 51% after 48 hours (Figure 3B). These results indicate that both Erg-GTr and Egr-GTr_2_ hyperpolarize the inner mitochondrial membrane. Additionally, to rule out if Erg itself (the non-azole fraction of the conjugates) could be responsible for this hyperpolarization, parasites were treated with this compound and ΔΨ_mit_ was monitored for up to 48 hours by flow cytometry. No differences were observed between control and Erg treated cells, indicating that hyperpolarization is a specific effect of both Erg-GTr hybrid molecules synthetized in this work.

### Erg-GTr and Erg-GTr_2_ treatment induce cell cycle alterations in promastigotes

**Figure 4:**
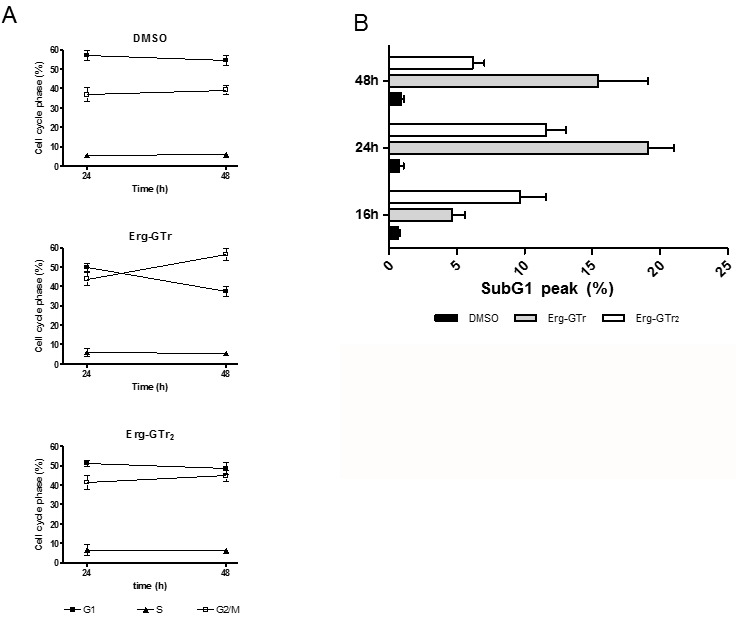
FIGURE 4: Analysis of the DNA content in *L. mexicana* promastigotes. Treated (1.4 μM Erg-GTr or 2.3 μM Erg-GTr_2_) or control cells (DMSO) were harvested at different time points and prepared for determination of DNA content. Acquisition was performed in arithmetic scale to obtain G1 and G2/M peaks and in logarithmic scale to earn the subG1 peak. **(A)** Percentage of cells in the different phases of the cell cycle after 24 and 48 hours treatment. **(B)** SubG1 peak in percentage after 16, 24 and 48 hours drug contact. Quantification was carried out using the WinMDI software. The bars represent the mean+/-S.D. of three individual cultures measured at each time point.

In order to analyze if Erg-GTr molecules influence the normal cell cycle distribution, the DNA content of treated cells was evaluated using propidium iodide. Results indicate a clear increase of cells in the G2/M phase after 48 hours, with the concomitant decrease of cells in the G1 phase (Figure 4A, Figure S1A). Differences were more obvious for parasites incubated with Erg-GTr, while those treated with Erg-GTr_2_ displayed a rather mild increase. In addition, histograms of treated cells as compared to control cells also showed an occurrence of cells in the SubG1 peak after 24 and 48 hours, which reveals DNA damage. DNA degradation reached almost 20% in treated cells, while control parasites displayed less than 1% (Figure 4B, Figure S1B).

### Ultrastructural alterations observed in promastigotes after Erg-GTr and Erg-GTr_2_ treatment

**Figure 5:**
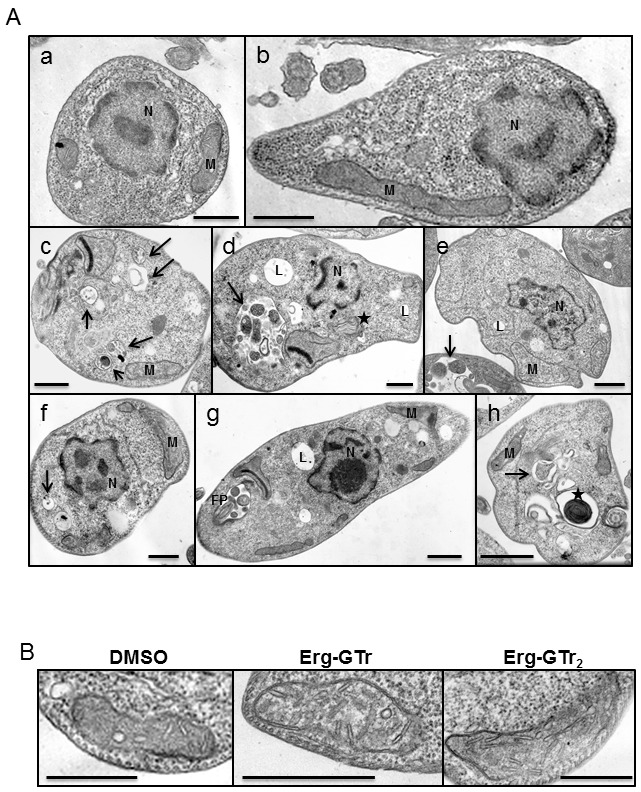
FIGURE 5: Transmission electron microscopy (TEM) of Epon-embedded cells. Parasites were treated with 1.4 μM Erg-GTr, 2.3 μM Erg-GTr_2_ or DMSO (control cells), harvested after 48 hours and prepared for TEM. **(A)** The most obvious alterations in Erg-GTr-treated cells are as follows: increase of vacuolar structures containing cytoplasmic material (c, d, f, h), myelin-like structures (d, h), increase of lysosomes (d, e, g), and flagellar pocket containing some material (g). Note the usual appearance of mitochondria, nucleus and cytoplasmic content in control cells (a, b). Abbreviations used: lysosome (L); nucleus (N); flagellar pocket (FP); mitochondrion (M). Arrows and asterisk indicate autophagosomes and myelin-like structures, respectively. **(B)** Mitochondrion zoom in to show cristae. More than 50% of the Erg-GTr treated parasites showed increase in cristae number and size. Bars represent 0.5 μ each.

To analyze if changes observed after parasite treatment with Erg-GTr or Erg-GTr_2_ can be associated with morphological alterations, cells were prepared for transmission electron microscopy as described in materials and methods section. As it can be seen in the figure 5A, treated parasites showed an evident increase of vacuolar structures containing cytoplasmic material (c, d, f, h) as compared with control parasites which were treated with the solvent (a, b).

Myelin-like structures (d, h), increase of lysosomes (d, e, g), and flagellar pocket containing some material (g) were also observed. Interestingly, the number and size of mitochondrion cristae was higher in Erg-GTr and Erg-GTr_2_ treated parasites (Figure 5B), which correlates with the hyperpolarization measured by flow cytometry.

### Erg-GTr and Erg-GTr_2_ trigger intracellular reactive oxygen species level and Ca^2+^ release in promastigotes

**Figure 6:**
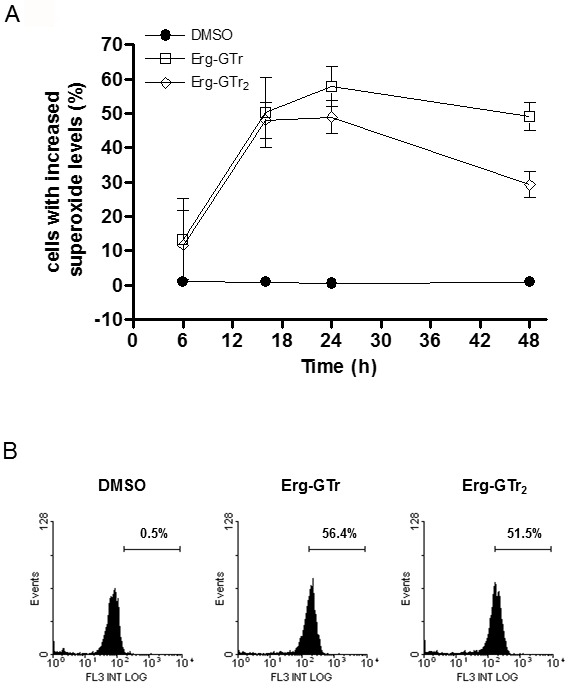
FIGURE 6: Superoxide production after Erg-GTr or Erg-GTr2 treatment. Parasites were treated with 1.4 μM Erg-GTr or 2.3 μM Erg-GTr_2_ or DMSO (control cells), harvested after different time points and then prepared to measure intracellular superoxide using dyhidroethidium. **(A)** Superoxide production kinetics in control and treated parasites. Results represent mean +/- SD of three independent experiments. **(B)** Representative FACS histograms showing the superoxide-sensitive fluorescence of control and treated promastigotes after 24 hours incubation. The percentages were determined using the WinMDI software.

To evaluate the intracellular level of reactive oxygen species (ROS) after Erg-GTr or Erg-GTr_2_ treatment, parasites were incubated with dihydroethidium, which become oxidized in the presence of the superoxide anion to form the fluorescent DNA-intercalating agent ethidium, i.e. the emitted fluorescence is proportional to the amount of ROS present. Figure 6A represents a ROS production kinetics analysis performed between 6 and 48 hours post-treatment with both molecules. The ROS level in Erg-GTr mono- or di-substituted treated parasites was significantly higher as in control cells, reaching a maximum after 24 hours, when more than half of the population showed increased intracellular ROS level (Figure 6B). In fact, ROS levels increased already during the first 6 hours of treatment when compared with control cells, which maintained a ROS level lower than 2% throughout the whole kinetic study.

**Figure 7:**
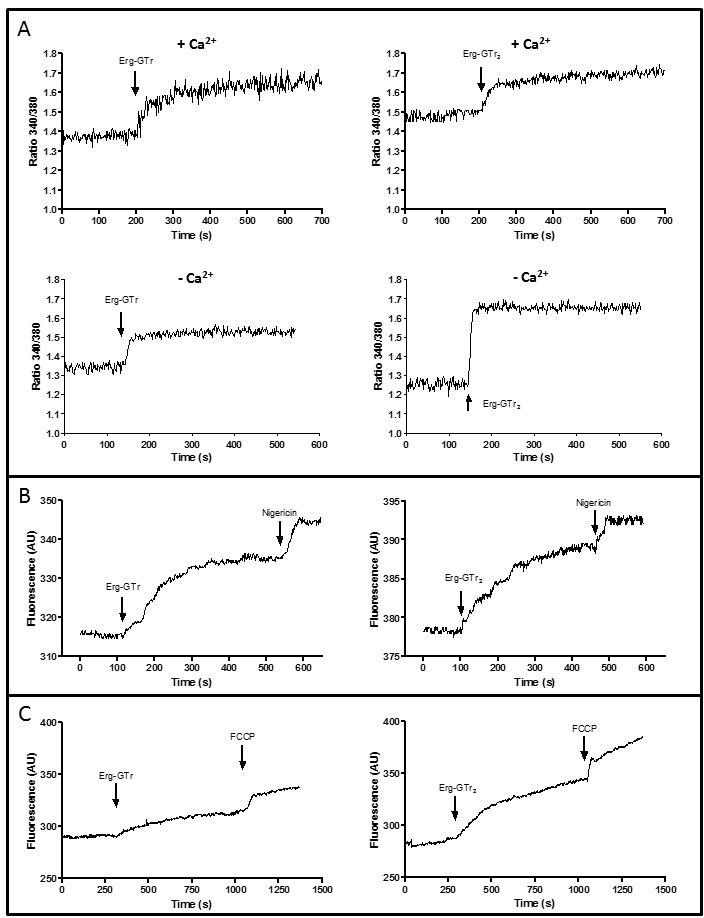
FIGURE 7: Analysis of the effect of Erg-GTr and Erg-GTr_2_ on the cytoplasmic Ca^2+^ concentration. Stationary phase parasites were loaded with Fura-2AM, acridine orange, or rhodamine 123 to analyze, immediately after addition of Erg-GTr (1.4 μM) or Erg-GTr_2_ (2.3 μM), the changes in the cytoplasmic Ca^2+^ concentration, acidocalcisome alkalinization, and mitochondrion membrane potential, respectively. **(A)** Relative Ca^2+^ concentration given as normalized OD_340/380 _ratio in the presence or absence of extracellular Ca^2+^. Arrows indicate addition of Erg-GTr or Erg-GTr_2_. **(B)** Effect of ergosterone-triazol hybrid molecules on the alkalinization of acidocalcisomes. The excitation wavelength was 488 nm, and the emission wavelength was 530 nm. Nigericin (2 μM) was used as control to complete organelle alkalinization. **(C)** Relative rhodamine 123 fluorescence before and after addition of Erg-GTr or Erg-GTr_2_. FCCP (1 μM) was employed as control for total mitochondrion depolarization. Fluorescence was also register at 488 nm excitation and 530 nm emission. All graphics are representative charts of one of at least three experiments that showed essentially the same results.

In order to evaluate the possible effect of Erg-GTr and Erg-GTr_2_ on the cytoplasmic Ca^2+^ concentration ([Ca^2+^]_cyt_) in *L. mexicana* promastigotes, parasites were loaded with fura 2-AM, a fluorimetric Ca^2+^ indicator which allows the measurement of intracellular changes in its concentration. Figure 7A represents the relative [Ca^2+^]_cyt_, indicated as normalized OD_340/380 _ratio. Addition of Erg-GTr (1.4 μM) or Erg-GTr_2_ (2.3 μM) induced a rapid increase of the [Ca^2+^]_cyt_, reaching a plateau after a few minutes. Interestingly, when extracellular Ca^2+^ was completely sequestered by EGTA addition, the same result was obtained, indicating that Ca^2+^ was release from intracellular parasite organelles and not from the extracellular medium.

*Leishmania* can store Ca^2+^ in different compartments like the endoplasmic reticulum, the mitochondrion, and the acidocalcisomes 9. The major Ca^2+^ reservoir is represented by this last organelle 10. In order to analyze if the [Ca^2+^]_cyt_ increase is due to its release from acidocalcisomes, parasites were charged with acridine orange and assayed fluorimetrically. Since acridine orange accumulates in acidic compartments, it allows detection of alkalinization. Addition of Erg-GTr (1.4 μM) or Erg-GTr_2_ (2.4 μM) caused a rapid fluorescence increase, indicating organelle alkalinization (Figure 7B). In these experiments nigericin, a H^+^/K^+^ exchanger, was employed as a control, showing a further reduction of the acidity of these organelles, as expected.

As the *Leishmania* mitochondrion seems to be involved in [Ca^2+^]_cyt_ regulation and can also accumulate Ca^2+^ 9, the effect of Erg-GTr and Erg-GTr_2_ was analyzed immediately after its addition to parasites preloaded with rhodamine 123. Conversely to the results observed by flow cytometry after 8 hours of incubation with Erg-GTr or later, a slow fluorescence increase was detected when the hybrid molecules were added to rhodamine 123 pre-loaded parasites, indicating depolarization of mitochondrial membrane potential (Figure 7C). Therefore, it can be suggested that Erg-GTr and Erg-GTr_2_ can also induce, at least in part, Ca^2+^ release from the mitochondrion.

## DISCUSSION 

Sterols are essential components of cell membranes. Mammalian cells are able to synthetize cholesterol; the main sterol found in their membranes. By contrast, fungi and protozoa produce a special class of them, ergosterol, which is vital for parasitic growth and viability, but is absent from mammalian cell membranes 5,11. Azoles are widely known anti-fungals and anti-trypanosomal agents, because this family of compounds affects the ergosterol, but not appreciably the cholesterol biosynthesis 5,12. Consequently, new formulations based on ergosterol/azole chemotherapeutic agents for the treatment of human trypanosomiasis and leishmaniasis represent a plausible route for the discovery of new and well-suited drugs against these diseases.

In this work, ergosterol was oxidized to ergosterone to allow the coupling of triazol rings on the C3 and/or C6 positions of this sterol using Girard reagent as a linkage molecule.

The ergosterone-coupled triazol compounds displayed an antiproliferative effect against *Leishmania* *mexicana* promastigotes in a low micromolar range, were the highest toxic compounds evaluated, and showed the highest selectivity index (~10). In drug discovery for infectious diseases of the developing world, it has been established that a hit (chemical starting points) should have a selectivity index of at least 10 13. Therefore, Erg-GTr variants represent promising molecules, which could be further modified to improve their selectivity and action.

For a better understanding of the Erg-GTr and Erg-GTr_2_ effects on *Leishmania*, features of cell death mechanisms were evaluated. The Nomenclature Committee on Cell Death (NCCD) has proposed a new classification of cell death pathways based on biochemical and functional aspects. Accidental cell death (ACD) is produced by severe insults and is in effect immediate. By contrast, regulated cell death (RCD) is originated as an adaptive response that (unsuccessfully) attempt to restore cellular homeostasis and habitually happens in a relatively delayed mode 14,15. In our study, ACD was discarded, since PI staining demonstrated that plasma membrane integrity of *Leishmania* promastigotes was maintained for 24 hours drug contact, and only appeared long after apparition of others cell death markers. Conversely, RCD features, as phosphatidylserine exposure and DNA damage, start to emerge early. It was demonstrated that Erg-GTr_2_ and Erg-GTr induced a slight and moderate cell cycle arrest in the G2/M phase, respectively. Many compounds trigger cell cycle arrest in G2/M phase by causing DNA damage 16,17,18, and this phenomenon has also been reported in *Trypanosomatidae* 19,20. Since DNA degradation was observed prior to alteration of cell cycle progression, it may be hypothesized that *Leishmania* blockage in G2/M phase caused by both molecules could be related to DNA damage.

Ultra-structural changes in treated parasites were evident in TEM studies. The findings are consistent with a marked increase in autophagy. In previous studies, similar ultrastructural modifications were observed in *Leishmania *and* T. cruzi* treated with different drugs that affect the parasite’s sterol metabolism: BPQ-OH, ketoconazole and azasterol, i.e. inhibitors of squalene synthase, 14-α-demethylase, and δ-24-sterol methyltransferase, respectively 21,22,23. Induction of autophagy by Erg-GTr and Erg-GTr_2_ indicates a cell remodelling response to the deleterious effects on membrane structure and function. These compounds may exert their action, at least partially, by alteration of the membrane lipid composition, as it has been described for other azoles 5. Additionally, it can be speculated that the presence of Erg in their structures, which could be incorporated into membranes, may contribute to this effect.

*Leishmania* has only one prominent mitochondrion, which occupies approximately 12% of the protozoan body 9. This organelle constitutes an important target for sterol biosynthesis inhibitors, especially azoles and azasteroles, because ergosterol is an important component of the parasite’s mitochondrial membranes 5. Interestingly, Erg-GTr and Erg-GTr_2_ affected the parasite’s mitochondrion increasing mitochondrial cristae number and inducing organelle hyperpolarization. Mitochondrion hyperpolarization as a result of cellular injury had been occasionally described in the literature compared with many more publications documenting mitochondrial depolarization after cellular insults 24. Interestingly, hyperpolarization was preceded by a slight reduction of mitochondrial membrane potential immediately after Erg-GTr and Erg-GTr_2_ addition as evidenced by the rhodamine 123 assay. To us, the most plausible interpretation of the data is that the hyperpolarization is a compensatory response to the slight but constant effect of Erg-GTr and Erg-GTr_2_ on the Ψ_mit_. To the best of our knowledge, the effect caused by Erg-triazol coupled molecules on parasite mitochondrion is a new characteristic of azole derivative activity, as azoles action reported does not maintain the mitochondrion integrity and functionality 12,21,25. Probably, the different effect found is due to, at least in part, some differential interference with the ergosterol synthesis. Additionally, it can also be expected that Erg-triazol hybrid molecules show a different mechanism of action, based on the novel structure.

ROS molecules have been well-documented to play an important role in signal cascades in numerous physiological events 26,27 and have also been widely reported to participate in signal transduction related to cell death mechanisms 8, even in *Trypanosoma* and *Leishmania* parasites 28,29,30,31. Remarkably, ROS production was, along with hyperpolarization, the first event observed in Erg-GTr/Erg-GTr_2_ treated cells. The analogous behaviour of ΔΨ_mit_ and ROS suggests their direct relationship. Incubation with the ROS scavenger N-acetyl-cysteine did not prevent mitochondrion hyperpolarization, but could revert partially ROS formation, indicating that hyperpolarization is not a consequence of the ROS action but may be their source (data not shown). It is well known that the mitochondrion is the most important ROS producer at the cellular level. Electron leakage in the electron transport chain during cellular respiration results in superoxide production 32. As it is expected, hyperpolarization may depict a higher mitochondrion activity, which traduces in a higher flow through electron transport chain that could be responsible for the increased superoxide level found. However, additional ROS sources cannot be discarded.

ROS production could explain some of the phenotypes observed in Erg-GTr/Erg-GTr_2_ treated parasites, especially DNA damage, augmentation of autophagy, and PS exposure. All of them have been reported for member of the *Trypanosomatidae *family as a consequence of ROS action 28,29,33. An interesting example, which resembles ours findings, was reported in *Leishmania infantum*. Promastigotes of this parasite subjected to heat shock stress underwent apoptotic-like cell death mediated by ROS. The parasite showed a mitochondrial hyperpolarization which was demonstrated to lead to ROS production 29.

Alteration of the intracellular Ca^2+^ homeostasis has been suggested as a promising target strategy against *Leishmania *and *Trypanosoma*. Amiodarone, an antiarrhythmic drug, and the azol, posaconazole, are able to increase the [Ca^2+^]_cyt_ and inhibit sterol synthesis in these parasites 34. It has been reported that treatment with amiodarone (in combination with itraconazole) or posaconazole, led to the cure of patients with Chagas’ disease 35,36. In our work, parasites exposed to Erg-GTr or Erg-GTr_2_ rapidly released important amounts of Ca^2+^ to the cytosol. It could be demonstrated that Ca^2+^ release came from intracellular compartments since measurements were also performed in the absence of extracellular Ca^2+^. According to the rhodamine 123 and acridine orange assay, increase in [Ca^2+^]_cyt_ proceeds slightly from the mitochondrion but mainly from acidocalcisomes.

**Figure 8:**
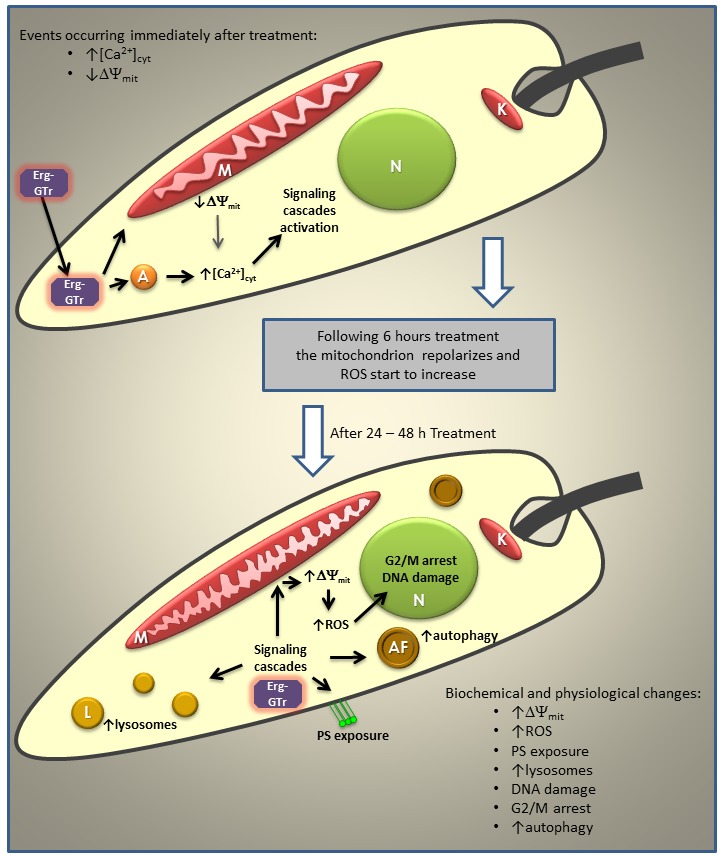
FIGURE 8. Model of the events sequence induced in *L. mexicana *promastigotes after addition of Erg-GTr or Erg-GTr_2_. When promastigotes are treated with ergosterone-triazol coupled molecules, a rapid increase in the cytoplasmic Ca^2+^ level, which mainly comes from acidocalcisomes, is induced. This divalent cation should activate signal cascades that trigger some of the phenotypes observed as PS exposure, ↑ΔΨ_mit_, and autophagy exacerbation. Intracellular ROS increase is also one of the earliest events found after Erg-GTr or Erg-GTr_2_ addition. Hyperpolarized mitochondrion may originate large amounts of ROS. This effector can induce DNA damage, among others, which may conduce to cell cycle arrest. Finally, autophagy could also be directly induced by the effect of ergosterone-triazol coupled molecules on cell membranes, which has been widely reported in the literature to be caused by azoles. Abbreviations used: acidocalcisomes (A); autophagy vacuoles (AF); lysosomes (L); nucleus (N); kinetoplast (K); mitochondrion (M).

Taken together, our results demonstrated that ergosterone-triazol hybrid molecules induce several physiological and biochemical changes in *Leishmania mexicana* promastigotes including mitochondrion alteration, Ca^2+^ release from acidocalcisomes, autophagosome formation, intracellular ROS increase, phosphatidylserine exposure, among others (see model in Figure 8), which lead to parasite cell death. Ruling out the induction of accidental cell death and according to the recommendations of NCCD, we conclude that Erg-GTr and Erg-GTr_2_ trigger a regulated cell death process. Therefore, Erg-GTr represents a reasonable candidate to initiate studies with the view of developing new chemotherapeutic agents for the treatment of leishmaniasis.

## Materials and Methods

### Chemistry

Solvents were of analytical grade and acquired from Aldrich or Riedel-de-Haën. All reactions were routinely checked by TLC using Machery-Nagel alumina plates; spots were examined under UV light at 254 nm and further visualized by sulphuric acid-anisaldehyde spray. Column chromatography was performed on alumina (Merck). NMR spectra were recorded at 270 MHz for H^1^ and 67.5 MHz for C^13^ on a JEOL ECLIPSE 270 spectrometer. Chemical shifts were expressed in ppm (δ) relative to residual solvent signals and the coupling constants J are given in Hertz (Hz). IR and UV spectrums were performed on an IR-Force 200 Infrared Spectrometer Analyzer (Richen-force) and on a Nicolet Evolution 300 spectrophotometer (Thermo Electron Corporation). Melting points (mp) were taken uncorrected on a Sybron-Thermolyne MP-12615 apparatus.

#### Ergosterone (Erg) synthesis

Ergosterol was purchased from Sigma Chemical Co. (St. Louis, MO). Erg was synthetized by ergosterol (2.55 x 10^-4^ mol) oxidation using the Jones reactive (1.53 x 10^-3^ mol chromic anhydride, 1 mL sulfuric acid in 5 mL distilled water) 6. Solution was maintained for 12 hours under shaking at room temperature, followed by dehydration with isopropanol for 2 hours. Product was filtered and purified by column chromatography on alumina using 9.7:0.3 dichloromethane/methanol.

#### Girard-Triazol (GTr) synthesis

Triazol was purchased from Sigma Chemical Co. (St. Louis, MO). The Girard reagent GTr was synthetized dissolving Triazol (7.5 x 10^-3^ moles) in ethanol (20 mL) and mixed with ethyl chloroacetate (Aldrich) (9.4 x 10^-3^ moles). The reaction mixture was stirred in reflux at 90°C for 72 hours and then was settled on ice and mixed with hydrazine hydrate (2.9 x 10^-2^ moles) under agitation for 20 minutes. The solvent was evaporated in an ice bath, the residue was recrystallized in methanol, and solid purified by column chromatography on alumina.

#### Ergosterone-Girard/triazol (Erg-GTr) synthesis

Equimolar amounts of Erg and GTr were dissolved in methanol and stirred in reflux at 90°C for 15 hours. Thereafter, solvent was evaporated, the residue washed with acetone, and filtered by fluted. Following acetone evaporation, the solid was purified by column chromatography on alumina.

#### Ergosterone-Girard/trimethylethylammonium (Erg-GT) and ergosterone-Girard/pyridinium (Erg-GP) synthesis 

Girard reagents T and P (GT and GP) were obtained commercially from Aldrich. Synthesis of the corresponding Erg derivative molecule was performed as follows; equimolar amounts of Erg and GT or GP were dissolved in methanol and stirred in reflux at 90°C for 20 hours. Thereafter, solvent was evaporated, the residue washed with acetone, and filtered by fluted. After acetone evaporation, the reaction product was purified by column chromatography on alumina. In all cases di-substituted derivatives were obtained by mixing Girard reagents and ergosterone in a 2:1 molar ratio, respectively.

### Cell cultures

Promastigotes of *Leishmania mexicana* Bel 21 strain were cultured at 26°C in Liver Infusion Tryptone (LIT) medium supplemented with 10% fetal bovine serum (Internegocios). Primary cultures of human dermic fibroblasts were performed at 37°C in DMEN medium (Sigma) supplemented with 10% fetal bovine serum in a CO_2_ incubator at 5% CO_2_. 

### Determination of the half-inhibitory concentration

To determine the IC_50_ in parasites and human fibroblasts the protocol described by 37 was followed. Briefly, a suspension of 2 x 10^6^ parasites per mL^-1^ or 5 x 10^3^ fibroblasts per well were transferred into 96 well plates and different concentrations of the compounds were added. Plates were incubated under the corresponding culture conditions for 48 or 72 hours for *L. mexicana* or human fibroblasts, respectively. Thereafter, parasites plates were centrifuged prior to the elimination of the supernatant. Cell viability was measured by the modified MTT method, where cells were incubated with the MTT substrate at 0.2 mg x mL^-1^ for 4 hours. Colored crystals formed were solubilized in DMSO and absorbance was measured at 570 nm. IC_50_ was obtained from a dose response curve using the software OriginLab Pro 6.0^©^.

### Annexin V binding assay

The annexin V-AlexaFluor 488 (Invitrogen) was used following the manufacture recommendations. Briefly, 1 x 10^6^ parasites were washed with PBS and incubated with annexin V and propidium iodide (0.05 mg x mL^-1^) in 100 μL binding buffer for 15 minutes. Final volume was adjusted at 500 μL and samples were immediately analyzed in a Beckman Coulter Gallios Cytometer. 

### Mitochondrial membrane potential analysis

Mitotracker CMX Red(Invitrogen) was used to determine the mitochondrial membrane potential (Ψ_mit_) of treated parasites. After 6, 16, 24, and 48 hours treatment, cells were incubated in culture medium with 50 nM Mitotracker CMX Red for 30 minutes at 26°C in the dark. Thereafter, parasites were washed twice with PBS, resuspended in 1 mL buffer, and analyzed using a cytometer (Beckman Coulter). Parasites incubated with valinomycin (500 nM for 30 minutes) were used as positive control 38. To evaluate the effect of ergosterone coupled molecules on the mitochondrial membrane potential immediately after molecule addition we used the fluorophore rhodamine-123, a mitochondrion-specific cationic dye, which distributes across the inner mitochondrial membranes strictly according to their membrane potential, as described before 34. Briefly, 1 x 10^7^ parasites were washed twice with rhodamine 123 loading buffer (20 mM Tris-HCl, 130 mM KCl, 1 mM MgCl_2_, and 2 mM KH_2_PO_4_) and incubated with 20 μM rhodamine 123 for 45 minutes at 29°C under agitation. Samples were washed twice, resuspended in 500 μL of the same buffer, and transferred to a quartz cuvette. Fluorescence (ex 488 nm and em 530 nm) was registered in a Hitachi F-7000 Spectrofluorimeter. FCCP was used as control for mitochondrial membrane depolarization 39. 

### DNA content analysis

Propidium iodide was used to determinate the DNA content in parasites after treatment 38. 1 x 10^6^ parasites were washed with PBS and resuspended in 100 μL lysis buffer (7.7 mM Na_2_HPO_4_, 2.3 mM KH_2_PO_4_) containing 6 μM digitonin. Samples were incubated at 4°C for 30 minutes. Finally, 400 μL staining buffer (20 μg/mL propidium iodide in PBS) was added to each sample. Samples were kept on ice in the dark until their analysis in the cytometer (Beckman Coulter). 

### Transmission electron microscopy (TEM)

For TEM, 10^8^ parasites were fixed in 2% (vol/vol) glutaraldehyde in 0.2 M sodium cacodylate buffer containing 0.12 M sucrose for 1 h at 4°C. Thereafter samples were washed four times (10 min each) and stored overnight in sodium cacodylate buffer. Before dehydration in ethanol, cells were postfixed in osmium tetroxide (1.5%, wt/vol) and stained in 0.5% uranyl acetate. Samples were cleared in propylene oxide and embedded in Agar 100 as described by 40. Sections were stained in 5% (wt/vol) uranyl acetate and 0.4% (wt/vol) lead citrate. 

### Reactive oxygen species determination

Dihydroethidium (Invitrogen) was used to detect the intracellular level of superoxide anion. The fluorescent indicator was used following the manufacture recommendations with some modifications to adapt it for *Leishmania* parasites. After parasite treatment, aliquots from each culture were taken and incubated with DE (2.5 μM final concentration) for 30 minutes at 26°C. Thereafter, samples were washed with PBS and immediately analyzed in a Beckman Coulter Cytometer. 

### Cytoplasmic Ca^2+^ level measurements

The fluorescent ratiometric Ca^2+^ indicator Fura 2-AM was used to analyze intracellular Ca^2+^ concentration 41. Promastigotes from stationary phase cultures were washed twice with PBS containing 1% glucose and incubated for 3 hours at 29°C with 4 μM Fura 2-AM, 2.4 μM probenecid, and 0.05% pluronic acid. Thereafter, parasites were washed twice, resuspended, and placed in a stirred quartz cuvette at 29°C. Fluorescence (ex 340 nm/380 nm and em 510 nm) was registered in a Hitachi F-7000 Spectrofluorimeter. Digitonin and EGTA were used to obtain the maximum and minimum [Ca^2+^] in the sample, respectively 41.

### Alkalinization of acidocalcisomes

To evaluate the effect of ergosterone coupled molecules on the Ca^2+^ reservoir organelle acidocalcisome, parasites were stained with acridine orange as described previously 42. Briefly, 1 x 10^7^ parasites were washed twice with loading buffer (20 mM Tris-HCl, 130 mM KCl, 1 mM MgCl_2_, and 2 mM KH_2_PO_4_) and incubated for 5 minutes with acridine orange (5 μM). Samples were washed twice, resuspended in 500 μL of the same buffer, and transferred to a stirred quartz cuvette at 29°C. Fluorescence (ex 488 nm and em 530 nm) was registered in a Hitachi F-7000 Spectrofluorimeter. Nigericin was used as control for acidocalcisome alkalinization.

## SUPPLEMENTAL MATERIAL

Click here for supplemental data file.

All supplemental data for this article are also available online at http://microbialcell.com/researcharticles/ergosterone-coupled-triazol-molecules-trigger-mitochondrial-dysfunction-oxidative-stress-and-acidocalcisomal-ca2-release-in-leishmania-mexicana-promastigotes/.

## References

[1] DNDi_Leishmaniasis_factsheet (2014). Leishmaniasis: A Global Disease with Regional
Challenges..

[2] TDR (2015). WHO estimates investments needed for neglected tropical
diseases..

[3] Bhattacharya SK, Sinha PK, Sundar S, Thakur CP, Jha TK, Pandey K, Das VR, Kumar N, Lal C, Verma N, Singh VP, Ranjan A, Verma RB, Anders G, Sindermann H, Ganguly NK (2007). Phase 4 trial of miltefosine for the treatment of Indian visceral
leishmaniasis.. J Infect Dis.

[4] Weete JD, Abril M, Blackwell M (2010). Phylogenetic distribution of fungal sterols.. PLoS ONE.

[5] De Souza W, Rodrigues JCF (2009). Sterol Biosynthesis Pathway as Target for Anti-trypanosomatid
Drugs.. Interdiscip Perspect Infect Dis.

[6] Bowden K, Heilbron IM, Jones ERH, Weedon BCL (1946). 13..

[7] Girard A, Sandulesco G (1936). Sur une nouvelle série de réactifs du groupe carbonyle, leur
utilisation à l’extraction des substances cétoniques et à la caractérisation
microchimique des aldéhydes et cétones.. HCA.

[8] Kroemer G, Galluzzi L, Vandenabeele P, Abrams J, Alnemri ES, Baehrecke EH, Blagosklonny MV, El-Deiry WS, Golstein P, Green DR, Hengartner M, Knight RA, Kumar S, Lipton SA, Malorni W, Nuñez G, Peter ME, Tschopp J, Yuan J, Piacentini M, Zhivotovsky B, Melino G, Nomenclature Committee on Cell Death 2009 (2009). Classification of cell death: recommendations of the Nomenclature
Committee on Cell Death 2009.. Cell Death Differ.

[9] Benaim G, Garcia CRS (2011). Targeting calcium homeostasis as the therapy of Chagas’ disease
and leishmaniasis - a review.. Trop Biomed.

[10] Docampo R, Huang G (2015). Calcium signaling in trypanosomatid parasites.. Cell Calcium.

[11] Garcia-Ruiz C, Mari M, Colell A, Morales A, Caballero F, Montero J, Terrones O, Basañez G, Fernández-Checa JC (2009). Mitochondrial cholesterol in health and disease.. Histol Histopathol.

[12] De Macedo-Silva ST, de Souza W, Fernandes Rodrigues JC (2015). Sterol biosynthesis pathway as an alternative for the
anti-protozoan parasite chemotherapy.. Curr Med Chem.

[13] Katsuno K, Burrows JN, Duncan K, Hooft van Huijsduijnen R, Kaneko T, Kita K, Mowbray CE, Schmatz D, Warner P, Slingsby BT (2015). Hit and lead criteria in drug discovery for infectious diseases
of the developing world.. Nat Rev Drug Discov.

[14] Galluzzi L, Vitale I, Abrams JM, Alnemri ES, Baehrecke EH, Blagosklonny MV, Dawson TM, Dawson VL, El-Deiry WS, Fulda S, Gottlieb E, Green DR, Hengartner MO, Kepp O, Knight RA, Kumar S, Lipton SA, Lu X, Madeo F, Malorni W, Mehlen P, Nuñez G, Peter ME, Piacentini M, Rubinsztein DC, Shi Y, Simon H-U, Vandenabeele P, White E, Yuan J (2012). Molecular definitions of cell death subroutines: recommendations
of the Nomenclature Committee on Cell Death 2012.. Cell Death Differ.

[15] Galluzzi L, Bravo-San Pedro JM, Vitale I, Aaronson SA, Abrams JM, Adam D, Alnemri ES, Altucci L, Andrews D, Annicchiarico-Petruzzelli M, Baehrecke EH, Bazan NG, Bertrand MJ, Bianchi K, Blagosklonny MV, Blomgren K, Borner C, Bredesen DE, Brenner C, Campanella M, Candi E, Cecconi F, Chan FK, Chandel NS, Cheng EH, Chipuk JE, Cidlowski JA, Ciechanover A, Dawson TM, Dawson VL (2015). Essential versus accessory aspects of cell death: recommendations
of the NCCD 2015.. Cell Death Differ.

[16] Wang JY, Cho SK (2004). Coordination of repair, checkpoint, and cell death responses to
DNA damage.. Adv Protein Chem.

[17] Wang HC, Pao J, Lin SY, Sheen LY (2012). Molecular mechanisms of garlic-derived allyl sulfides in the
inhibition of skin cancer progression.. Ann N Y Acad Sci.

[18] Guo J, Wu G, Bao J, Hao W, Lu J, Chen X (2014). Cucurbitacin B induced ATM-mediated DNA damage causes G2/M cell
cycle arrest in a ROS-dependent manner.. PLoS ONE.

[19] Valenciano AL, Ramsey AC, Mackey ZB (2015). Deviating the level of proliferating cell nuclear antigen in
Trypanosoma brucei elicits distinct mechanisms for inhibiting proliferation
and cell cycle progression.. Cell Cycle.

[20] Uzcátegui NL, Carmona-Gutiérrez D, Denninger V, Schoenfeld C, Lang F, Figarella K, Duszenko M (2007). Antiproliferative effect of dihydroxyacetone on Trypanosoma
brucei bloodstream forms: cell cycle progression, subcellular alterations,
and cell death.. Antimicrob Agents Chemother.

[21] Lorente SO, Rodrigues JCF, Jiménez Jiménez C, Joyce-Menekse M, Rodrigues C, Croft SL, Yardley V, de Luca-Fradley K, Ruiz-Pérez LM, Urbina J, de Souza W, González Pacanowska D, Gilbert IH (2004). Novel azasterols as potential agents for treatment of
leishmaniasis and trypanosomiasis.. Antimicrob Agents Chemother.

[22] Rodrigues JCF, Urbina JA, de Souza W (2005). Antiproliferative and ultrastructural effects of BPQ-OH, a
specific inhibitor of squalene synthase, on Leishmania
amazonensis.. Exp Parasitol.

[23] Santa-Rita RM, Lira R, Barbosa HS, Urbina JA, de Castro SL (2005). Anti-proliferative synergy of lysophospholipid analogues and
ketoconazole against Trypanosoma cruzi (Kinetoplastida: Trypanosomatidae):
cellular and ultrastructural analysis.. J Antimicrob Chemother.

[24] Perry SW, Norman JP, Barbieri J, Brown EB, Gelbard HA (2011). Mitochondrial membrane potential probes and the proton gradient:
a practical usage guide.. BioTechniques.

[25] Vannier-Santos MA, Urbina JA, Martiny A, Neves A, de Souza W (1995). Alterations induced by the antifungal compounds ketoconazole and
terbinafine in Leishmania.. J Eukaryot Microbiol.

[26] Khan AU, Wilson T (1995). Reactive oxygen species as cellular messengers.. Chem Biol.

[27] Wang X, Fang H, Huang Z, Shang W, Hou T, Cheng A, Cheng H (2013). Imaging ROS signaling in cells and animals.. J Mol Med.

[28] Figarella K, Uzcategui NL, Beck A, Schoenfeld C, Kubata BK, Lang F, Duszenko M (2006). Prostaglandin-induced programmed cell death in Trypanosoma brucei
involves oxidative stress.. Cell Death Differ.

[29] Alzate JF, Arias AA, Moreno-Mateos D, Alvarez-Barrientos A, Jiménez-Ruiz A (2007). Mitochondrial superoxide mediates heat-induced apoptotic-like
death in Leishmania infantum.. Mol Biochem Parasitol.

[30] Getachew F, Gedamu L (2012). Leishmania donovani mitochondrial iron superoxide dismutase A is
released into the cytosol during miltefosine induced programmed cell
death.. Mol Biochem Parasitol.

[31] Ribeiro GA, Cunha-Júnior EF, Pinheiro RO, da-Silva SAG, Canto-Cavalheiro MM, da Silva AJM, Costa PRR, Netto CD, Melo RCN, Almeida-Amaral EE, Torres-Santos EC (2013). LQB-118, an orally active pterocarpanquinone, induces selective
oxidative stress and apoptosis in Leishmania amazonensis.. J Antimicrob Chemother.

[32] Jastroch M, Divakaruni AS, Mookerjee S, Treberg JR, Brand MD (2010). Mitochondrial proton and electron leaks.. Essays Biochem.

[33] Chowdhury S, Mukherjee T, Chowdhury SR, Sengupta S, Mukhopadhyay S, Jaisankar P, Majumder HK (2014). Disuccinyl betulin triggers metacaspase-dependent endonuclease
G-mediated cell death in unicellular protozoan parasite Leishmania
donovani.. Antimicrob Agents Chemother.

[34] Benaim G, Sanders JM, Garcia-Marchán Y, Colina C, Lira R, Caldera AR, Payares G, Sanoja C, Burgos JM, Leon-Rossell A, Concepcion JL, Schijman AG, Levin M, Oldfield E, Urbina JA (2006). Amiodarone has intrinsic anti-Trypanosoma cruzi activity and acts
synergistically with posaconazole.. J Med Chem.

[35] Paniz-Mondolfi AE, Pérez-Alvarez AM, Lanza G, Márquez E, Concepción JL (2009). Amiodarone and itraconazole: a rational therapeutic approach for
the treatment of chronic Chagas’ disease.. Chemotherapy.

[36] Pinazo M-J, Espinosa G, Gállego M, López-Chejade PL, Urbina JA, Gascón J (2010). Successful treatment with posaconazole of a patient with chronic
Chagas disease and systemic lupus erythematosus.. Am J Trop Med Hyg.

[37] Novoa ML, Escalante Y, Maldonado L, Galindo-Castro I, Álvarez A, Figarella K, Marsiccobetre S, Arocha I, Nieves J, Salazar F, Gámez C, Canudas N, Tropper E, González T, Villamizar JE (2015). Synthesis and biological evaluation of
(-)-13,14-dihydroxy-8,11,13-podocarpatrien-7-one and derivatives from
(+)-manool.. Nat Prod Res.

[38] Figarella K, Rawer M, Uzcategui NL, Kubata BK, Lauber K, Madeo F, Wesselborg S, Duszenko M (2005). Prostaglandin D2 induces programmed cell death in Trypanosoma
brucei bloodstream form.. Cell Death Differ.

[39] Benaim G, Bermudez R, Urbina JA (1990). Ca2+ transport in isolated mitochondrial vesicles from Leishmania
braziliensis promastigotes.. Mol Biochem Parasitol.

[40] Glauert AM, Butterworth AE, Sturrock RF, Houba V (1978). The mechansim of antibody-dependent, eosinophil-mediated damage
to schistosomula of Schistosoma mansoni in vitro: a study by phase-contrast
and electron microscopy.. J Cell Sci.

[41] Philosoph H, Zilberstein D (1989). Regulation of intracellular calcium in promastigotes of the human
protozoan parasite Leishmania donovani.. J Biol Chem.

[42] Benaim G, Casanova P, Hernandez-Rodriguez V, Mujica-Gonzalez S, ParraGimenez N, Plaza-Rojas L, Concepcion JL, Liu Y-L, Oldfield E, Paniz-Mondolfi A, Suarez AI (2014). Dronedarone, an amiodarone analog with improved antiLeishmania
mexicana efficacy.. Antimicrob Agents Chemother.

