# Supplementary material for: Ergosterone-coupled Triazol molecules trigger mitochondrial dysfunction, oxidative stress, and acidocalcisomal Ca2+ release in Leishmania mexicana promastigotes
Source: Microb Cell. 2015 Dec 11;3(1):14–28. doi: 10.15698/mic2016.01.471 (PMC5354587; doi:10.15698/mic2016.01.471)
Supplement: Supplementary file 1 [file mic-03-014-s01.pdf]

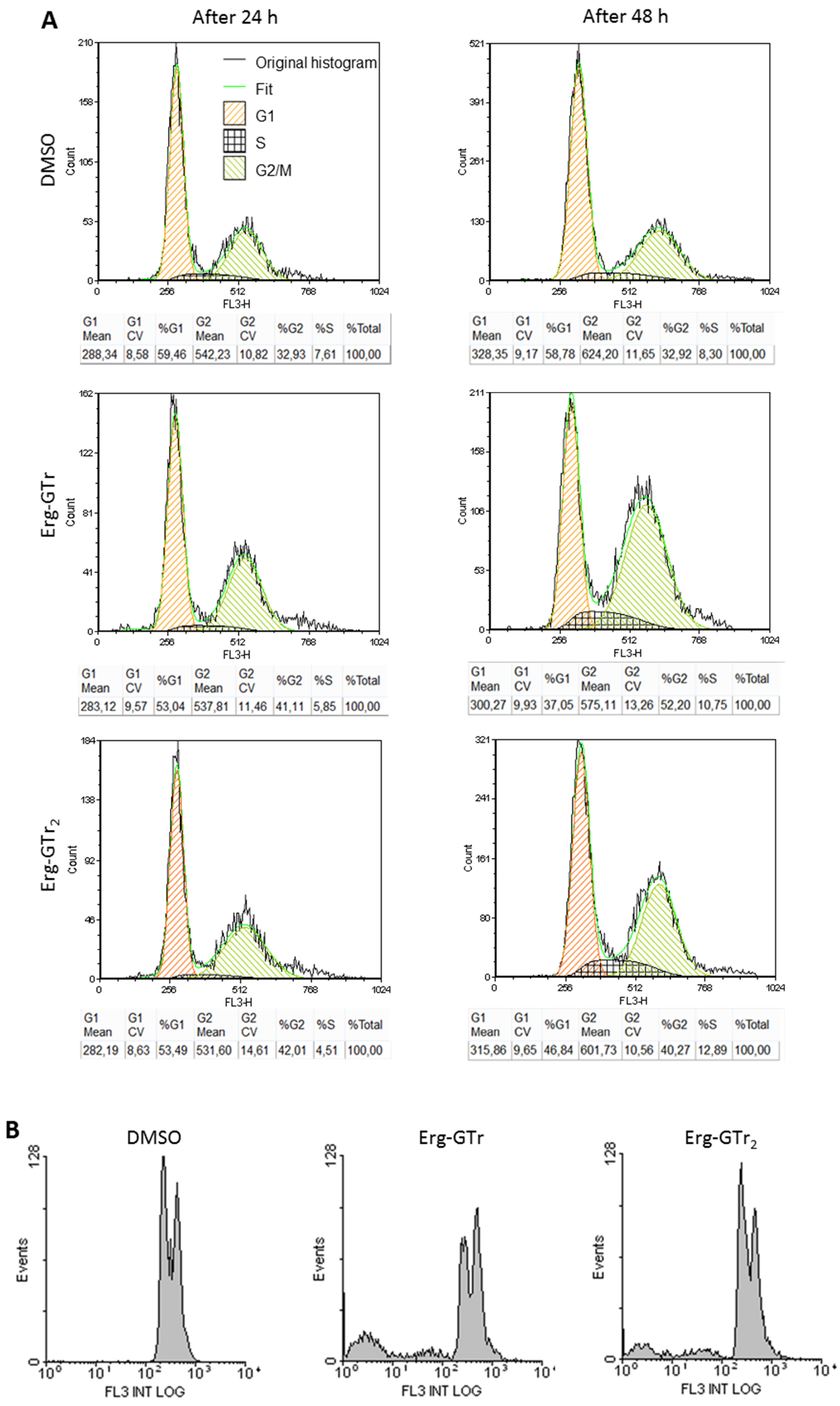

DMSO, after 24 h:

G1 Mean	G1 CV	%G1	G2 Mean	G2 CV	%G2	%S	%Total
288,34	8,58	59,46	542,23	10,82	32,93	7,61	100,00

DMSO, after 48 h:

G1 Mean	G1 CV	%G1	G2 Mean	G2 CV	%G2	%S	%Total
328,35	9,17	58,78	624,20	11,65	32,92	8,30	100,00

Erg-GTr, after 24 h:

G1 Mean	G1 CV	%G1	G2 Mean	G2 CV	%G2	%S	%Total
283,12	9,57	53,04	537,81	11,46	41,11	5,85	100,00

Erg-GTr, after 48 h:

G1 Mean	G1 CV	%G1	G2 Mean	G2 CV	%G2	%S	%Total
300,27	9,93	37,05	575,11	13,26	52,20	10,75	100,00

Erg-GTr2, after 24 h:

G1 Mean	G1 CV	%G1	G2 Mean	G2 CV	%G2	%S	%Total
282,19	8,63	53,49	531,60	14,61	42,01	4,51	100,00

Erg-GTr2, after 48 h:

G1 Mean	G1 CV	%G1	G2 Mean	G2 CV	%G2	%S	%Total
315,86	9,65	46,84	601,73	10,56	40,27	12,89	100,00

Supplemental figure 1. **Original histograms of the DNA content analysis.** A) Representative histograms obtained through events acquisition in arithmetic scale. Quantification was performed using the MultiCycle AV DNA analysis plug-in from the FCS Express 5 Plus Software. B) Representative histograms obtained when samples were acquired in logarithmic scale to visualize the subG1 peak.
